# The values of coagulation function in COVID-19 patients

**DOI:** 10.1371/journal.pone.0241329

**Published:** 2020-10-29

**Authors:** Xin Jin, Yongwei Duan, Tengfei Bao, Junjuan Gu, Yawen Chen, Yuanyuan Li, Shi Mao, Yongfeng Chen, Wen Xie

**Affiliations:** 1 Department of Laboratory Medicine, Zhongnan Hospital of Wuhan University, Wuhan, Hubei, China; 2 Department of Laboratory Medicine, Wuhan Leishenshan Hospital, Wuhan, Hubei, China; 3 Department of Laboratory Medicine, Wuhan Hospital of Traditional Chinese Medicine, Hubei, China; 4 Department of Laboratory Medicine, Wuhan Third Hospital, Hubei, China; 5 Medical Management Department, Zhongnan Hospital of Wuhan University, Wuhan, Hubei, China; 6 Medical Management Department, Wuhan Leishenshan Hospital, Wuhan, Hubei, China; Royal College of Surgeons in Ireland, IRELAND

## Abstract

**Objective:**

To investigate the blood coagulation function in COVID-19 patients, and the correlation between coagulopathy and disease severity.

**Methods:**

We retrospectively collected 147 clinically diagnosed COVID-19 patients at Wuhan Leishenshan Hospital of Hubei, China. We analyzed the coagulation function in COVID-19 patients through the data including thrombin-antithrombin complex (TAT), α2-plasmininhibitor-plasmin Complex (PIC), thrombomodulin (TM), t-PA/PAI-1 Complex (t-PAIC), prothrombin time (PT), international normalized ratio (INR), activated partial thromboplastin time (APTT), fibrinogen (FIB), thrombin time (TT), D-Dimer (DD), and platelet (PLT).

**Result:**

The levels of TAT, PIC, TM, t-PAIC, PT, INR, FIB, and DD in COVID-19 patients were higher than health controls (p<0.05), and also higher in the patients with thrombotic disease than without thrombotic disease (p<0.05). What’s more, the patients with thrombotic disease had a higher case-fatality (p<0.05). TAT, PIC, TM, t-PAIC, PT, INR, APTT, FIB, DD, and PLT were also found correlated with disease severity. Meanwhile, we found that there were significant difference in TAT, TM, t-PAIC, PT, INR, APTT, DD, and PLT in the death and survival group. Further using univariate and multivariate logistic regression analysis also found that t-PAIC and DD were independent risk factors for death in patients and are excellent predicting the mortality risk of COVID-19.

**Conclusion:**

Most COVID-19 patients with inordinate coagulation systems, dynamic monitoring of coagulation parameters might be a key in the control of COVID-19 death.

## 1. Introduction

People are going through a battle against novel coronavirus pneumonia (COVID-19) all over the world. By 3 April 2020, more than 1,010,000 people have been infected and more than 50,000 have died worldwide. The 2019 novel coronavirus (SARS-CoV-2) was confirmed as the pathogen of the COVID-19 and belongs to Beta coronavirus by the phylogenetic analysis, which is similar to severe acute respiratory syndrome coronavirus (SARS-CoV) and middle east respiratory syndrome coronavirus (MERS-CoV). Respiratory symptoms, fever, dry cough, and panting, even acute respiratory distress syndrome (ARDS) and acute cardiac injury manifested in the COVID-19 patients [1–4].

SARS-CoV-2 can cause a series of disorders of the coagulation systems including endothelial damage, coagulation activation, and intravascular fibrin deposition. For COVID-19 patients in severe, coagulation activation can be leading to the formation of thrombus and even disseminated intravascular coagulation (DIC) [5]. And the coagulation activation is more common in patients with pre-existing coagulation disorders. On the other side, although, most severe COVID-19 patients treated with extracorporeal membrane oxygenation (ECMO) to temporarily replaces cardiopulmonary function, the coagulation disorder is still one of the most common complications of ECMO, which is also a common cause of disruption of ECMO [6, 7]. Therefore, it is significant to monitor the coagulation systems parameters in COVID-19 patients to determine the coagulation function.

Previous studies have suggested that cytokine storms are typical abnormalities caused by SARS-CoV-2 [8, 9]. Nevertheless the underlying reason of the connection between the coagulation function and the disease severity was not completely clear. Especially, the index related thrombus such as thrombin-antithrombin complex (TAT), α2-plasmininhibitor-plasmin Complex (PIC), thrombomodulin (TM), t-PA/PAI-1 Complex (t-PAIC) are important markers in the process of venous thrombosis and significantly increased before thrombosis, which had not been reported. Therefore, this study was executed to evaluate the values of coagulation function for the prediction of severe COVID-19 patients.

## 2. Methods

### 2.1 Patients

We recruited 147 COVID-19 patients and 18 healthy controls in Wuhan Leishenshan Hospital, Hubei, China. The patients were diagnosed and classified followed the New Coronavirus Pneumonia Prevention and Control Program (7th edition) published by the National Health Commission of China. Briefly, the controls were hospital employees who underwent the healthy examination. The study was approved by the Ethics Committee of Zhongnan Hospital of Wuhan University.

#### 2.1.1. Standard of severe patients

Severe patients should have any of the following conditions:

- Respiratory distress, respiratory rate (RR) ≥ 30 times/minute;
- Under the resting state, the oxygen saturation ≤ 93%;
- Oxygen partial pressure (PaO2)/oxygen concentration (FiO2) in arterial blood ≤ 300 mmHg;
- Lung imaging showed obvious progression of the lesion >50% within 24–48 hours.

#### 2.1.2. Standard of critical patients

Critical patients should have any of the following conditions:

- Have respiratory failure and mechanical ventilation required;
- Shock;
- Complications of other organ failure require treatment in the intensive care unit (ICU).

### 2.2 Coagulation system examination

Fasting whole blood from every subjects was collected in an EDTA or Sodium Citrate anticoagulant treated tube and analyzed within 30 minutes of collection. Coagulation systems parameters, thrombin-antithrombin complex (TAT), α2-plasmininhibitor-plasmin Complex (PIC), thrombomodulin (TM), t-PA/PAI-1 Complex (t-PAIC), prothrombin time (PT), international normalized ratio (INR), activated partial thromboplastin time (APTT), fibrinogen (FIB), thrombin time (TT), D-Dimer (DD), and platelet (PLT) were analyzed.

### 2.3 Statistical analysis

Statistical analysis was performed with SPSS version 25.0 software and Graphpad Prism (version 7.0). All the measurement data were tested for normality, and non-normally distributed data were expressed as median (interquartile range), nonparametric Mann-Whitney test was used for comparison between the two groups. Univariate and multivariate analysis using logistic regression analysis: calculate the odds ratio and 95% confidence interval. The prediction of various indicators for prognosis COVID-19 patients were analyzed by the receiver operating characteristic (ROC) curves, and the area under the ROC curve (AUC) was measured to evaluate the predictive ability. Positive predictive and negative predictive value analysis by the MedCalc software. For all statistical analysis, P<0.05 was considered statistically significant.

## 3. Result

### 3.1 Clinical characteristics

Among the 147 COVID-19 patients, 76 were males and 71 were females, the median age was 64 years, while in the control group, 14 subjects were male and 4 subjects were females (S1 Table). There were 105 mild patients, 18 severe patients and 24 critical patients, of which 37 patients with thrombotic disease and 110 patients without thrombotic disease.

### 3.2 Coagulation parameters of healthy controls and COVID-19 patients

The levels of coagulation parameters at healthy controls and COVID-19 patients were demonstrated in Table 1 and S1 Table. As the Table 1 shown, more than 79% patients with the high level of the TAT, PIC, TM, t-PAIC than the normal level, and the positive rate of the TAT, PIC, TM, t-PAIC were significant differences in the healthy and COVID-19 groups. Although the patients with abnormal level of PT, APTT, FIB, TT, DD, PLT, were less than 50%, the positive rate of the PT, FIB, DD were significant differences in the healthy and COVID-19 groups. As the Table 1 shown, COVID-19 patients had significantly higher values of TAT, PIC, TM, t-PAIC, PT, INR, FIB, and DD than healthy controls, and there were no significant differences in APTT, TT, and PLT.

**Table 1:** Positive rate of the coagulation parameters between the healthy control and COVID-19 patients.

	Healthy control (n = 18)	COVID-19 patients (n = 147)	P value
**TAT**	16/18 (88.9%)	5/147 (3.4%)	**<0.001**
**Normal range <4 ng/mL**			
**Increased**	2/18 (11.1%)	142/147 (96.6%)	
**PIC**	17/18 (94.4%)	30/147 (20.4%)	**<0.001**
**Normal range <0.8 μg/mL**			
**Increased**	1 /18 (5.6%)	117/147 (79.6%)	
**TM**	18/18(100%)	29/147 (19.7%)	**<0.001**
**Normal range 3.8–13.3 TU/mL**			
**Increased**	0/18 (0%)	118/147 (80.3%)	
**t-PAIC**	18/18(100%)	19/147 (12.9%)	**<0.001**
**Normal range**			
**Female<10.5 g/mL; Male<17.0 ng/mL**			
**Increased**	0/18 (0%)	128/147 (87.1%)	
**PT**	18/18(100%)	103/147(70.0%)	**0.015**
**Normal range 9–13 s**			
**Increased**	0/18 (0%)	44/147 (30.0%)	
**INR**	18/18(100%)	103/147(70.0%)	**0.015**
**Normal range 0.76–1.24**			
**Increased**	0/18 (0%)	44/147 (30.0%)	
**APTT**	18/18(100%)	120/147 (81.6%)	0.09
**Normal range 20–40 s**			
**Increased**	0/18 (0%)	27/147 (18.4%)	
**FIB**	18/18(100%)	112/147 (76.2%)	**0.042**
**Normal range 2–4 g/L**			
**Increased**	0/18 (0%)	35/147 (23.8%)	
**TT**	18/18(100%)	143/147 (97.3%)	1.000
**Normal range 14–21 s**			
**Increased**	0/18 (0%)	4/147 (2.7%)	
**DD**	18/18(100%)	68/147 (46.3%)	**<0.001**
**Normal range <0.55 mg/L**			
**Increased**	0/18 (0%)	79/147 (53.7%)	
**PLT**	18/18(100%)	136/147 (92.5%)	0.483
**Normal range 125–350 10**^**9**^**/L**			
**Decreased**	0/18 (0%)	11/147 (7.5%)	

Abbreviations: IQR, interquartile range. TAT, Thrombin-Antithrombin complex; PIC, α2-plasmininhibitor-plasmin Complex; TM, Thrombomodulin; t-PAIC, t-PA/PAI-1 Complex; PT, prothrombin time; INR, international normalized ratio; APTT, activated partial thromboplastin time; FIB, fibrinogen; TT, thrombin time; DD, D-Dimer; PLT, platelet.

### 3.3 Coagulation parameters of COVID-19 patients with thrombotic diseases vs patients without thrombotic diseases

**Fig 1:**
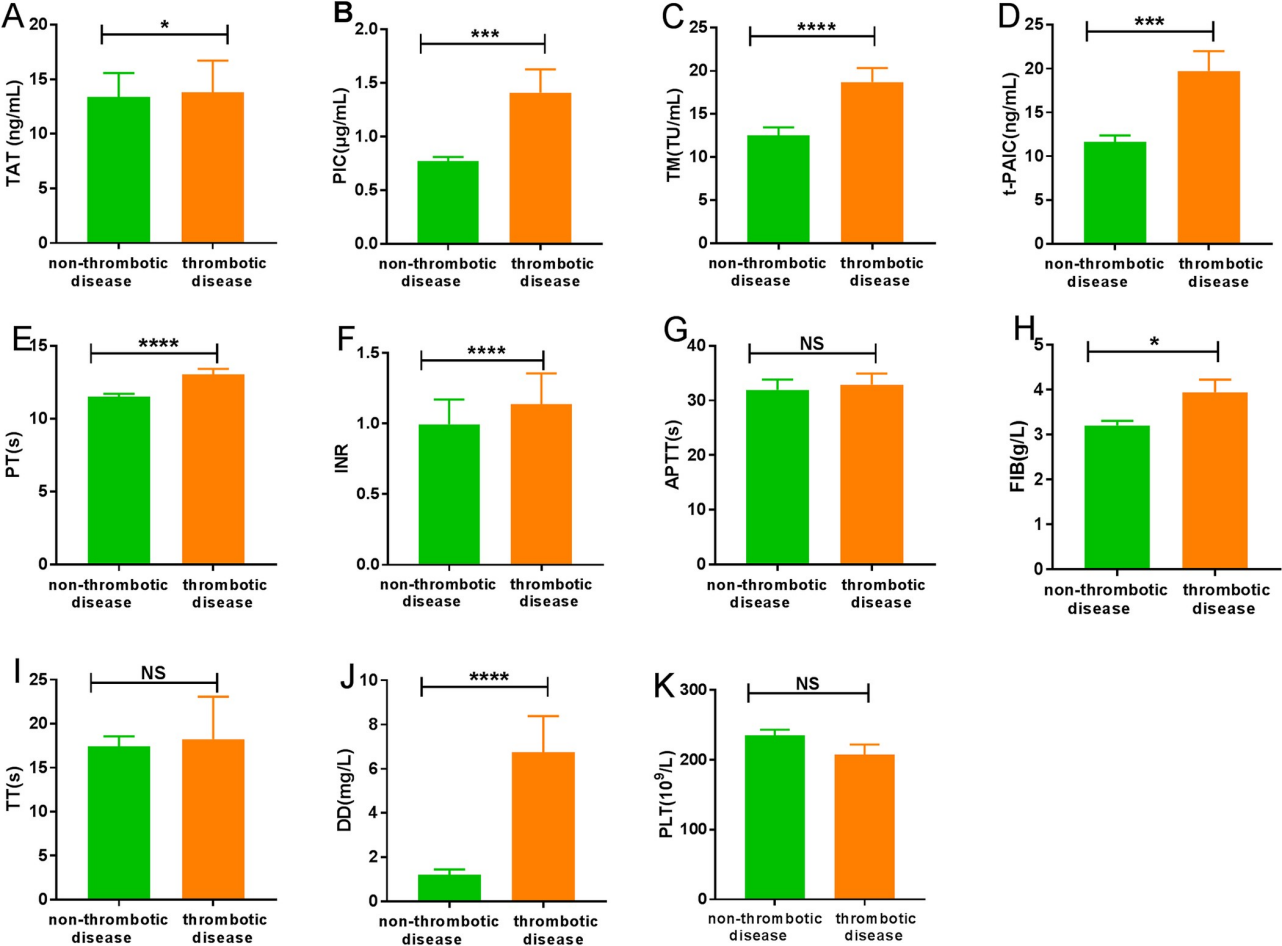
Characteristics of coagulation parameters for COVID-19 patients in non-thrombotic disease group and thrombotic disease group. (A) TAT; (B) PIC; (C) TM; (D) t-PAIC; (E) PT; (F) INR; (G) APTT; (H) FIB; (I) TT; (J) DD; (K) PLT. *P<0.05; ***P<0.001; ****P<0.0001; NS: P>0.05.

Among the 147 patients, 37 patients with thrombotic disease, 110 were without the thrombotic disease. There were 7 patients died among the thrombotic disease group, while 3 patients died in the non-thrombotic disease group (Table 2). The patients in thrombotic disease group had a higher case-fatality rate than the non-thrombotic disease group (p<0.05). The levels of coagulation parameters at the thrombotic disease group and non-thrombotic disease group were demonstrated in Fig 1. COVID-19 patients with thrombotic disease had significantly higher levels of TAT, PIC, TM, t-PAIC, PT, INR, FIB, and DD than those without the thrombotic disease. There were no significant differences in levels of APTT, TT, and PLT.

**Table 2:** Case-fatality rate in the thrombotic disease and non-thrombotic disease.

	Death	Survival	Total	Case-fatality rate
**Thrombotic disease**	7	30	37	18.9%
**Non-thrombotic disease**	3	107	110	2.7%
**Total**	10	137	147	6.8%

### 3.4 Coagulation parameters of three subgroups patients

**Fig 2:**
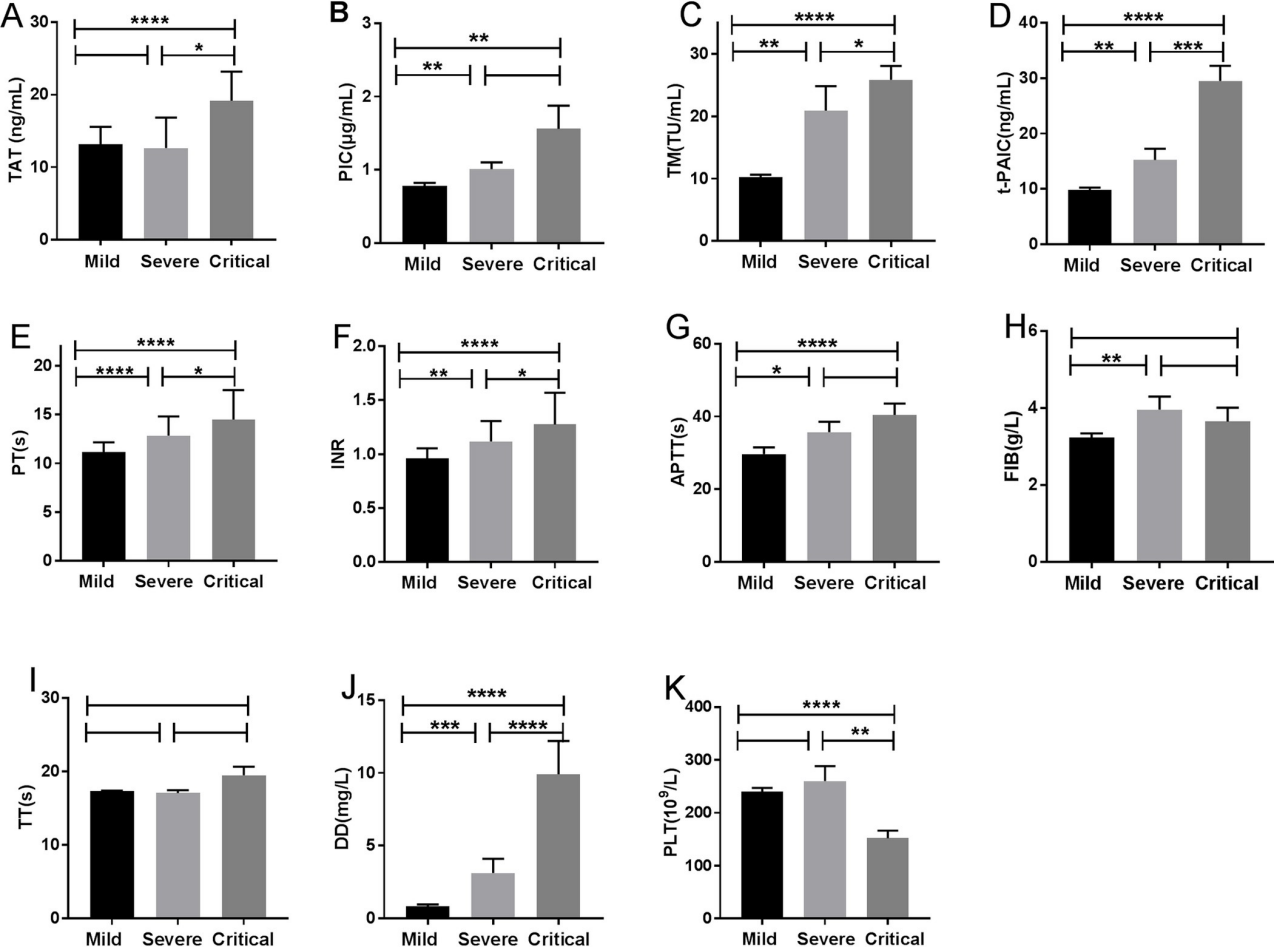
Characteristics of coagulation parameters among mild, severe and critical patients with COVID-19 pneumonia. (A) TAT; (B) PIC; (C) TM; (D) t-PAIC; (E) PT; (F) INR; (G) APTT; (H) FIB; (I) TT; (J) DD; (K) PLT. *P<0.05; **P<0.05; ***P<0.001; ****P<0.0001; NS: P>0.05.

**Fig 3:**
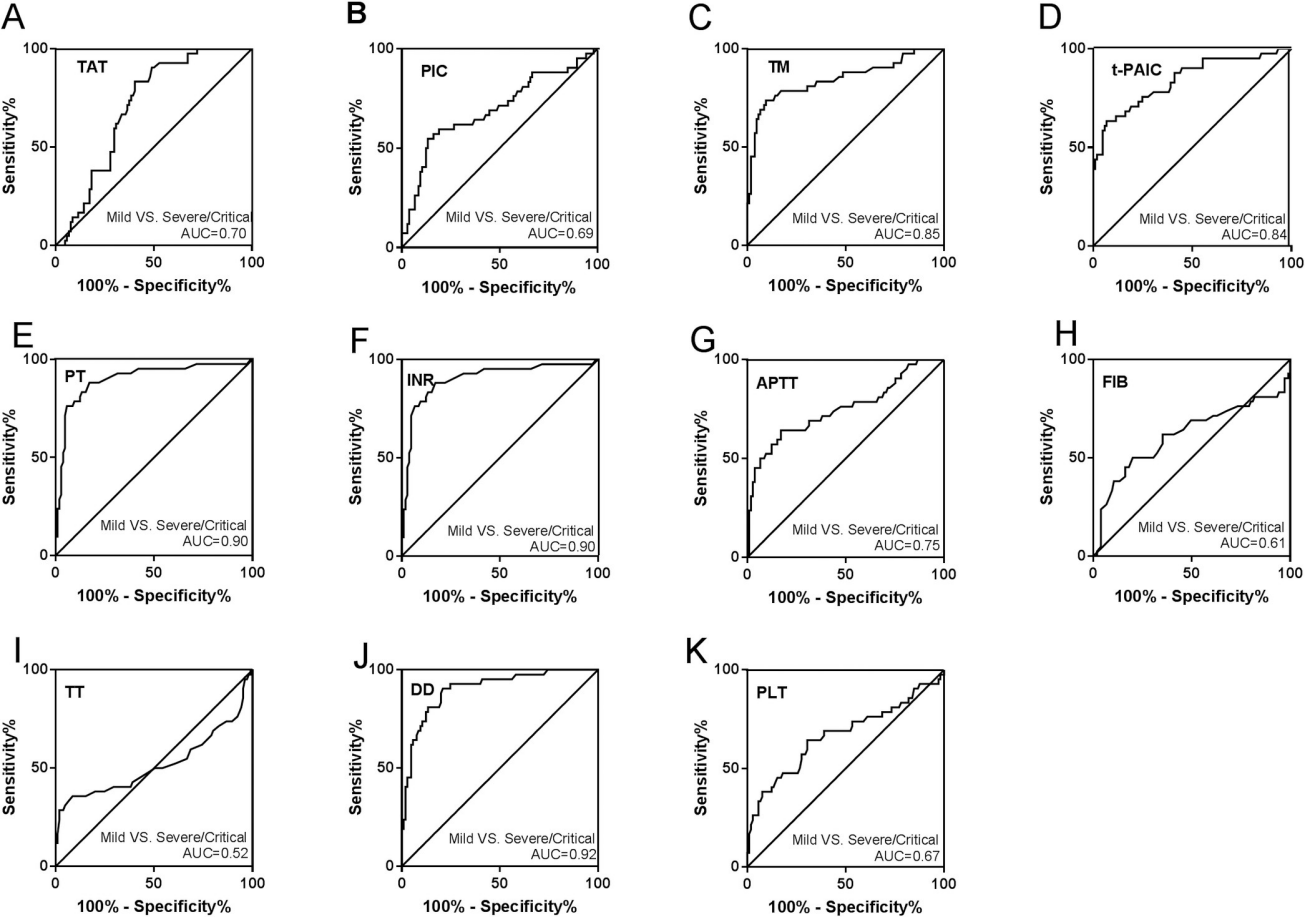
Receiver operator characteristic curves for coagulation parameters of COVID-19 patients in mild VS severe/critical. (A) TAT; (B) PIC; (C) TM; (D) t-PAIC; (E) PT; (F) INR; (G) APTT; (H) FIB; (I) TT; (J) DD; (K) PLT.

Among the 147 included patients, 105 patients belonged to mild group, 18 were severe, and 24 were critical. TM, t-PAIC, PT, INR, DD were significantly different among the three groups, while TT was no significant difference (Fig 2C–2F, 2I and 2J). The more severe in COVID-19 patients, the higher levels of TM, t-PAIC, PT, INR, and DD. TAT and PLT were significantly different between the severe and critical groups or mild and critical group, and no significance were found between the mild and severe groups (Fig 2A and 2K). With regard to PIC and APTT, there were significant difference between mild and severe or mild and critical patients, and no significant difference in severe and critical group (Fig 2B and 2G). In addition, there was a significant difference of FIB in mild group and severe group, however no significant difference was found between severe and critical group or mild and critical patients (Fig 2H). The ROC curves and AUC (Fig 3) demonstrated that TM (AUC = 0.85), t-PAIC (AUC = 0.84), PT (AUC = 0.90), and DD (AUC = 0.92) had good predictive ability in mild VS severe/critical (all p<0.05, Fig 3).

### 3.5 Coagulation parameters of COVID-19 patients in death and survival group

**Fig 4:**
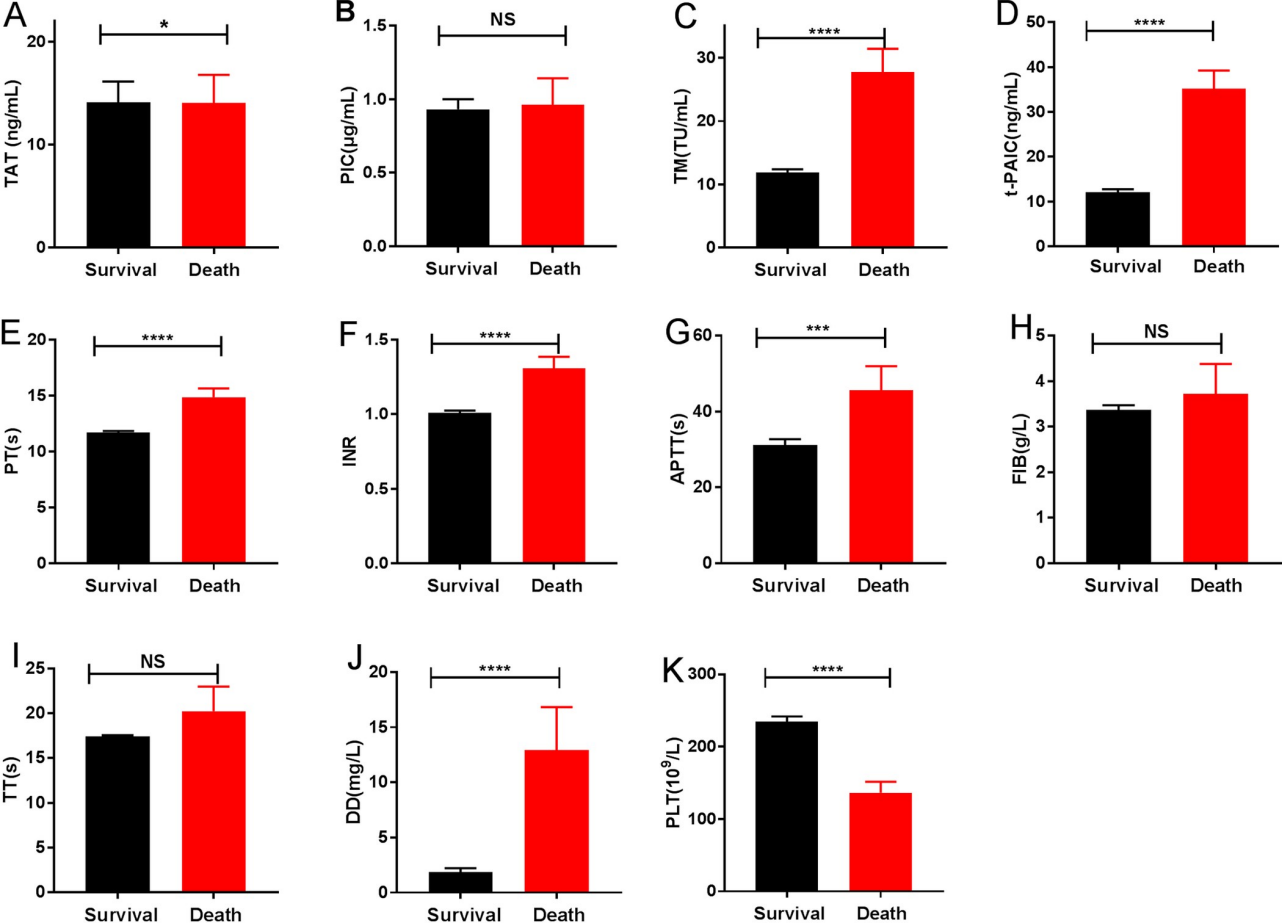
Characteristics of coagulation parameters in death and survival group. (A) TAT; (B) PIC; (C) TM; (D) t-PAIC; (E) PT; (F) INR; (G) APTT; (H) FIB; (I) TT; (J) DD; (K) PLT. *P<0.05; ***P<0.001; ****P<0.0001; NS: P>0.05.

**Fig 5:**
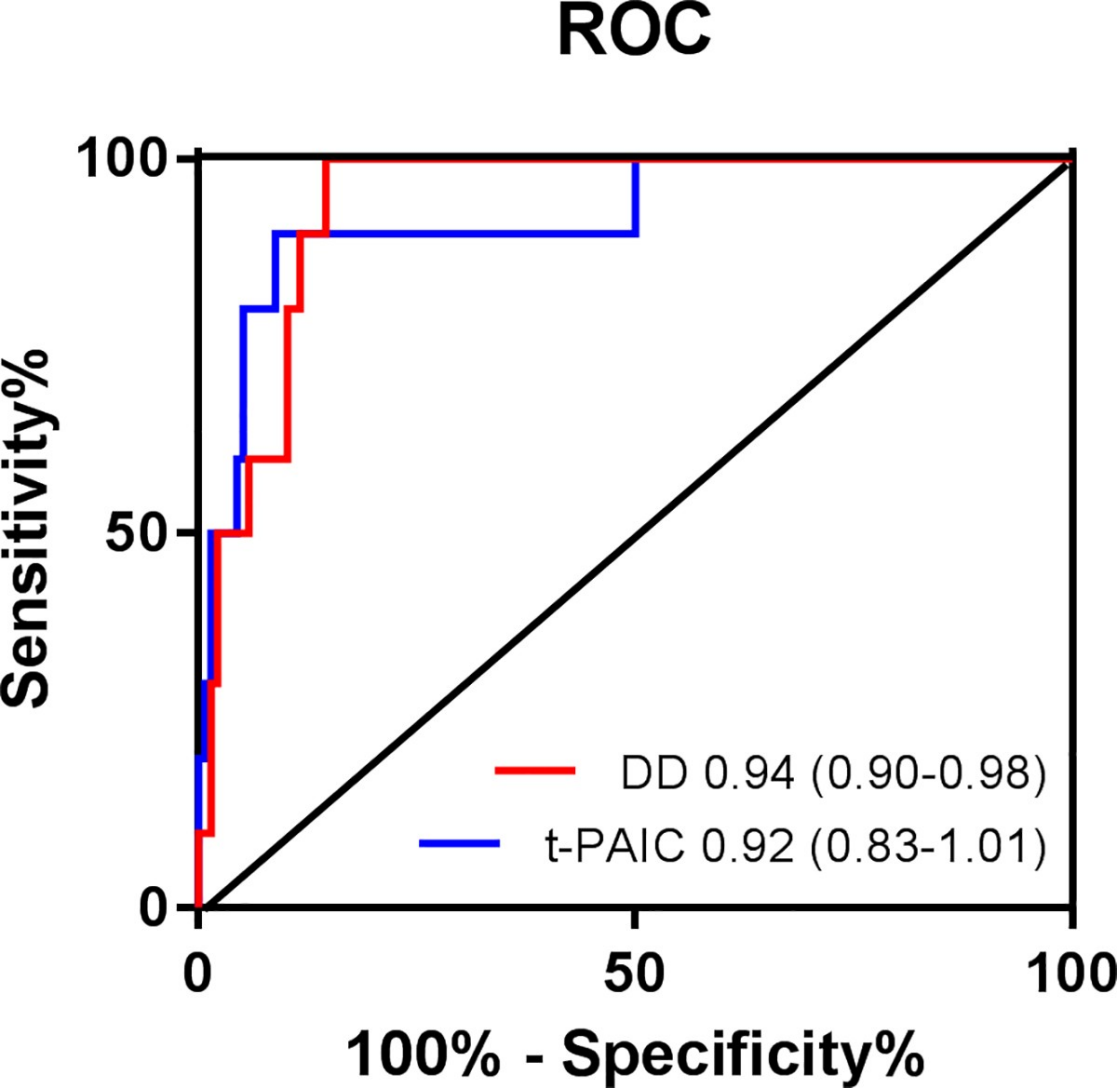
Receiver operator characteristics curves for coagulation parameters prediction of COVID-19 prognosis.

The levels of coagulation parameters at death and survival group were demonstrated in Fig 4. COVID-19 patients in the death group had significantly higher levels of TAT, TM, t-PAIC, PT, INR, APTT, and DD, but lower PLT level than the survival group. There were no significant differences in levels of TT, PIC, and FIB. Further analysis using univariate and multivariate logistic regression also found that t-PAIC and DD were independent risk factors for patients death (Table 3). ROC were drawn to analyze the prognostic values of coagulation parameters in COVID-19 patients, the AUC of t-PAIC and DD were 0.92 [95% confidence interval (95%CI) = 0.92–1.01], 0.94 [95%CI = 0.90–0.98] (Fig 5). It was showed that the t-PAIC and DD were excellent in independently predicting the mortality risk of COVID-19. The sensitivity and specificity of t-PAIC in predicting death respectively were 90.0% and 91.2% with the cut-off of greater than 20.6 ng/mL, and the positive predictive value and negative predictive value were 42.73% and 99.20%. And those for DD were 100.0% and 86.0% with the cut-off of greater than 2.78 mg/L, the positive predictive value and negative predictive were 34.26% and 100.00%.

**Table 3:** Univariate and multivariate logistic regression analyses for factors predictive of COVID-19 death.

Variables	Univariate analysis		Multivariate analysis	
	Odd Ratio (95% CI)	p value	Odd Ratio (95% CI)	p value
**Age**	1.077 (1.011–1.148)	**0.022**	1.113 (0.963–1.286)	0.146
**TAT**	1.000 (0.972–1.029)	0.995		
**PIC**	1.047 (0.490–2.236)	0.906		
**TM**	1.085 (1.037–1.136)	**<0.001**	1.053 (0.972–1.140)	0.206
**t-PAIC**	1.162 (1.088–1.240)	**<0.001**	1.148 (1.031–1.278)	**0.011**
**PT**	1.534 (1.183–1.989)	**0.001**	0.902 (0.565–1.441)	0.662
**APTT**	1.019 (0.997–1.040)	0.087		
**FIB**	1.203 (0.773–1.871)	0.413		
**TT**	1.205 (0.975–1.489)	0.085		
**DD**	1.158 (1.068–1.256)	**<0.001**	1.121 (1.009–1.244)	**0.033**
**PLT**	0.982 (0.971–0992)	**0.001**	0.990 (0.974–1.006)	0.202

## 4. Discussion

We reported the coagulation function of COVID-19 patients in Wuhan Leishenshan Hospital of Hubei, China. The world health organization had declared the COVID-19 was an international public health emergency at the end of January [10]. Within a period of four months, more than 1,010,000 people have been infected and more than 50,000 people have died worldwide. Although most of the COVID-19 patients have mild symptoms with good prognosis and the crude mortality rate was about 2.3%, for patients progressing to severe or critical, mortality rates have increased significantly with crude death rates reaching 49% for critically [11, 12]. Early identification of severe patients will help improve recovery rates and reduce mortality.

Studies have shown that COVID-19 patients can develop a variety of secondary diseases that cause pathological changes such as hematology, immunity, biochemistry, etc. [13, 14], but the changes in coagulopathy are not well understood. In this study, a retrospective analysis was conducted about the coagulation systems parameters of 147 patients. TAT, PIC, TM, t-PAIC, PT, INR, APTT, FIB, TT, DD, and PLT are expected to assess the function of the coagulation systems in patients and indicate the severity of the patients to provide clinical assistance.

COVID-19, as a life-threatening infectious disease, can cause endothelial damage, coagulation activation, and intravascular fibrin deposition. Results of this study indicated that compared with the health controls, the TAT, PIC, TM, t-PAIC, PT, INR, FIB, and DD higher in COVID-19, while the APTT, TT, and PLT with no difference. Meanwhile, they were also no significant differences in thrombotic disease group and the non-thrombotic disease group. While the levels of TAT, PIC, TM, t-PAIC, PT, INR, FIB, and DD in thrombotic disease group were higher than non-thrombotic disease group. Four items of thrombosis detection (TAT, PIC, TM and t-PAIC) can predict the possibility of thrombosis in the patients at an early stage. TAT as a marker for activation of the coagulation systems, to determine the optimal period of anticoagulation therapy and early diagnosis of thrombosis and pre-DIC [15, 16]. PIC as a marker of fibrinolytic system activation, predicting the formation of thrombus, assisting the diagnosis of DIC, and guiding antifibrinolytic treatment [17]. The elevated level of TM indicates an impaired vascular endothelial system [18, 19]. t-PAIC, key marker of fibrinolytic system, suggesting thrombus progression [20, 21]. Plasma levels of these sensitive biomarkers of endothelial injury in COVID-19 patients may be useful for evaluating the endothelial injury in COVID-19. Besides, we also found the COVID-19 patients with thrombotic disease had a higher case-fatality rate. Therefore, it is a situation that requires our vigilance when the COVID-19 patient with abnormal coagulation systems parameters, and it is likely to be accompanied by thrombosis, and the risk of death is greater.

TAT, PIC, TM, t-PAIC, PT, INR, APTT, FIB, DD, and PLT were also found correlated with disease severity, while the TT was no correlation (Fig 2). TT is the time for plasma to coagulate after adding a prothrombin solution in the plasma, and reflects the presence of anticoagulants. Based on clinical practice and ROC analysis between mild and non-mild patients (Fig 3), some cut-off values of the test items were obtained. With TM >13.65 TU/mL, DD>1.03 mg/L, progress to severe illness should be closely observed and prevented. Among them, the rise of DD concentration is most obvious, similar to Wang’s [22] and Guan [23] study. DD is elevated because inflammation causes coagulation activation. Our results suggested that patients’ coagulation parameters should be taken seriously to avoid progression of COVID-19. As reported, anticoagulant treatment is required in severe and critical patients, and DD ≥ 5 μg/mL is used as an indicator to adjust anticoagulant therapy, while improving the prognosis [24]. To reverse intravascular coagulation, microthrombi formation, fibrin deposition, COVID-19 patients might need anticoagulant or fibrinolytic therapy.

Further, we found the COVID-19 patients in death group had significantly higher levels of TAT, TM, t-PAIC, PT, INR, APTT, and DD than the survival group, and the PLT decreased. It is shown that the patients have thrombocytopenia, elevated level of DD, and a prolonged APTT, suggesting that COVID-19 patients death may be associated with DIC [25, 26]. Most severe COVID-19 patients develop dyspnea or hypoxemia one week after the onset, and their lung is more serious and gas exchange cannot be performed. When ventilator cannot restore lung function, ECMO has become an effective treatment to rescue critical patients, but it is only temporarily replacing cardiopulmonary function, and there are still many complications. Among them, the coagulation systems disorder is still one of the most common complications of ECMO, which terminate the ECMO [27, 28]. Therefore, safe and scientific monitoring of the ECMO loop and thrombosis in patients, then early intervention can further reduce the harm of these complications to promote effective circulation support for ECMO. TAT, PIC, TM, and t-PAIC are the indicators of early prediction and monitoring of DIC, and DD is the indicator that a thrombus is occurring or ongoing. What’s more, our findings demonstrated that the prediction of the t-PAIC and DD can be as the excellent independently predicting the mortality risk of COVID-19, with an AUC of 0.92, 0.94, respectively (Fig 5).

Coagulation systems have values in COVID-19 patients because most patients have coagulopathy. Preventing recognition or blocking the occurrence of thrombus or DIC in COVID-19 patients, dynamic monitoring of coagulation systems parameters might be a key in the control of COVID-19 death.

## Supporting information

S1 Table Comparison of coagulation parameters between COVID-19 patients and healthy control. (DOC)
