# Supplementary material for: The values of coagulation function in COVID-19 patients
Source: PLoS One. 2020 Oct 29;15(10):e0241329. doi: 10.1371/journal.pone.0241329 (PMC7595402; doi:10.1371/journal.pone.0241329)
Supplement: S1 Table — (DOC) [file pone.0241329.s001.doc]

**S1 Table**. Comparison of coagulation parameters between COVID-19 patients and Healthy control

|  | **Healthy control (n=18)** | **COVID-19 patients (n=147)** | **P value** |
| --- | --- | --- | --- |
| **Age, median (IQR), y** | 43 (34-51) | 64 (54-73) |  |
| **Gender (Female/Male)** | 4/14 | 71/76 |  |
| **TAT** | 1.70 (0.90-2.50) | 5.4 (2.9-14.5) | **<0.001** |
| **PIC** | 0.41 (0.27-0.51) | 0.70 (0.54-1.10) | **<0.001** |
| **TM** | 7.85 (6.90-9.18) | 10.00 (8.20-15.20) | **<0.001** |
| **t-PAIC** | 5.95 (3.98-8.18) | 10.80 (7.10-14.85) | **<0.001** |
| **PT** | 10.9 (10.48-11.33) | 11.30 (10.70-12.50) | **0.021** |
| **INR** | 0.93 (0.90-0.97) | 0.97 (0.92-1.08) | **0.019** |
| **APTT** | 27.15 (25.23-29.13) | 28.50 (24.95-32.80) | 0.105 |
| **FIB** | 2.71 (2.48-2.98) | 3.00 (2.62-3.90) | **0.029** |
| **TT** | 17.55 (17.08-18.13) | 17.30 (16.65-18.05) | 0.252 |
| **DD** | 0.15 (0.12-0.24) | 0.72 (0.29-2.29) | **<0.001** |
| **PLT** | 270.50 (201.25-290.00) | 214.00 (178.50-265.5) | 0.083 |
| Abbreviations: IQR, interquartile range. TAT, Thrombin-Antithrombin complex; PIC, α2-plasmininhibitor-plasmin Complex; TM, Thrombomodulin; t-PAIC, t-PA/PAI-1 Complex; PT, prothrombin time; INR, international normalized ratio; APTT, activated partial thromboplastin time; FIB, fibrinogen; TT, thrombin time; DD, D-Dimer; PLT, platelet. | | | |
